# Advances in the multi-omics landscape of follicular lymphoma

**DOI:** 10.7150/ijbs.80401

**Published:** 2023-03-27

**Authors:** Tianyuan Xu, Zhong Zheng, Weili Zhao

**Affiliations:** Shanghai Institute of Hematology, State Key Laboratory of Medical Genomics, National Research Center for Translational Medicine at Shanghai, Ruijin Hospital Affiliated to Shanghai Jiao Tong University School of Medicine, Shanghai, China

**Keywords:** follicular lymphoma, genomics, transcriptomics, microenvironment, biomarkers, targeted therapy, immunotherapy

## Abstract

Follicular lymphoma (FL) is the most common indolent lymphoma originating from germinal center B cells. FL represents a clinically and biologically heterogeneous disease. Most patients have favorable outcomes, but a subset of patients experiences early progression or transformation and has a poor prognosis. Abnormalities in FL cells and tumor microenvironment have been revealed using multi-omics techniques, including genomic, epigenomic, transcriptomic and proteomic analysis. Recurrent somatic gene aberrations mainly involve epigenetic modifiers, transcription factors, oncogenic pathways and microenvironment modulators. Single-cell transcriptomic analysis show marked inter- and intra-patient FL subclone heterogeneity. In addition, a comprehensive profile of microenvironmental components is provided, unveiling the crosstalk between tumor and microenvironment that induce FL progression and facilitate immune escape. Together, these studies provide insights into the mechanisms and biomarkers of high-risk FL populations, as well as the potential targeted and immunotherapy options. Future research should focus on integrating multi-omics aberrations to optimize therapeutic strategies in FL.

## Introduction

Follicular lymphoma (FL) is the second most common type of non-Hodgkin's lymphoma, accounting for approximately 15-25% of adult lymphomas with an annual incidence of 3-5/100 000 1, 2. As an indolent lymphoma, about 80% of FL patients have an overall survival (OS) of more than 10 years, but most patients experience recurrent remission and relapse with increasing drug resistance 3. Moreover, a subset of patients experiences early progression or transformation and has a poor prognosis 4, 5. The underlying genetic, epigenetic, and microenvironmental mechanisms driving FL progression and resistance need to be further elucidated.

The pathogenesis of FL is a multistep process. The initial and hallmark genetic aberration in FL is the t (14;18)(q32;q21) translocation detected in >85% of patients. This occurs during V(D) J recombination of pro/pre B cells in the bone marrow, leading to sustained expression of the anti-apoptotic gene BCL2. Then naïve B cells enter the germinal center (GC) region of lymph nodes, where they undergo activation-induced cytidine deaminase (AID)-mediated somatic hypermutation (SHM) and class switch recombination (CSR). The overexpression of BCL2 provides a survival advantage for GC which is independent of BCR affinity 6. These BCL2(+) FL-like cells traffic between secondary lymphoid organs and bone marrow, accumulating additional genetic alterations through repeated GC reentry, and eventually develop into overt FL 7.

FL exhibits a high dependence on the tumor microenvironment (TME) composed of different types of immune cells and stromal cells. In lymph nodes, the FL microenvironment mainly consists of CD4+ T follicular helper (Tfh) cells, CD4+ T regulatory cells (Tregs), CD8+ cytotoxic T cells (CTLs), macrophages, follicular dendritic cells (FDCs) and fibroblastic reticular cells (FRCs) 8, 9. Through various soluble factors and ligand-receptor interactions, FL-TME crosstalk supports tumor cell survival and proliferation, promotes immune escape, and also serves as promising prognostic biomarker and therapeutic target 10.

The multi-omics approaches provide a comprehensive assessment of the biological features of disease by integrating information at different levels, including the genome, epigenome, transcriptome and proteome 11, 12. These techniques have been applied to not only comprehensively elucidate the pathogenesis and progression of the disease, but also to identify the biomarkers for improving clinical management and explore novel treatment approaches. Here, this review discusses the multi-omics abnormalities in tumor cells and TME of FL, and their application in risk stratification and treatment approaches.

## Multi-omics aberrations of FL

Through whole-genome sequencing, targeted deep sequencing and single-nucleotide polymorphism array, mutations and chromosomal structural variants have been identified (Table 1) 13-22.

### Epigenetic modifiers

Mutations in epigenetic modifiers are key pathogenic factors of FL. These include histone modifiers (KMT2D, CREBBP/EP300, and EZH2), members of the linker histone H1 family (HIST1H1C and HIST1H1E), and SWI/SNF chromatin remodeling complex genes (ARID1A and BCL7A). These mutations inhibit critical transcriptional programs for B cell selection and differentiation, and are detected in almost all FL patients.

KMT2D (also called MLL2) is the most common mutation detected in 70-90% of patients. Inactivating mutations of KMT2D result in reduced lysine 4 of histone H3 (H3K4) methylation in FL cells, altering the expression of a wide range of genes including CD40, JAK-STAT, Toll-like receptor, B cell receptor signaling pathways and tumor suppressor genes 23. Early loss of KMT2D during B cell development promotes proliferation and malignant outgrowth of B cells 24. CREBBP and its paralogue EP300 are mutated in 50-70% and 10-20% of patients respectively. Loss-of-function mutations in CREBBP cause focal depletion of enhancer H3K27 acetylation, resulting in transcriptional repression of genes regulating cell differentiation, BCR/CD40 signaling and MHC presentation 25, 26. CREBBP-deficient hematopoietic stem and progenitor cells are unable to acetylate P53 and exhibit a defective DNA damage response, thereby facilitating the accumulation of subsequent mutations 27. EP300 regulates transcriptional programs that partially overlap with CREBBP, and deletion of both produces synthetic lethal effects 28. Histone-lysine N-methyltransferase EZH2 is the catalytic subunit of the PRC2/EED-EZH2 complex and increases H3K27 trimethylation to silence gene transcription 29. Mutations in EZH2 are observed in 20-30% of patients. Gain-of-function mutations in EZH2 repress the expression of proliferation checkpoint CDKN1A and differentiation-related gene PRDM1 and IRF4 30, 31.

Inactivating mutations in the linker-histone H1 family members and SWI/SNF chromatin remodeling complex genes are observed in 28-44% and 11-15% of patients respectively. These tumor suppressors organize chromatin structure by regulate DNA-ribosome topology and histone modifications 32, 33. The combined deletion of H1c/H1e in mice can enhance the self-renewal properties of GC B cells through focal chromatin relaxation and stem cell gene upregulation 32.

### Transcription factors

Approximately half of FL patients harbor non-silent mutations in lymphoid transcription factors such as BCL6, MEF2B and FOXO1^34^. BCL6 is mutated or translocated in 20% of patients. BCL6 is a transcriptional repressor of DNA damage checkpoints and B cell differentiation genes, and can cooperate with histone modifiers through recruiting histone demethylases and deacetylases 35, 36. In FL, highly expressed BCL6 drives the survival and growth of FL cells in part through repressing NOTCH pathway genes 37, 38. MEF2B, a transcriptional activator of BCL6 and MYC, is mutated in 7-15% of patients. Mutations in MEF2B disrupt its interaction with the corepressor CABIN1 and reduce the sensitivity to inhibitory signals, resulting in increased transcriptional activity 39, 40. Gain-of-function mutations in FOXO1 are detected in 12% of patients, leading to hyperactivation of PI3K and SAPK/JNK signaling 41.

### Oncogenic pathways

**Figure 1:**
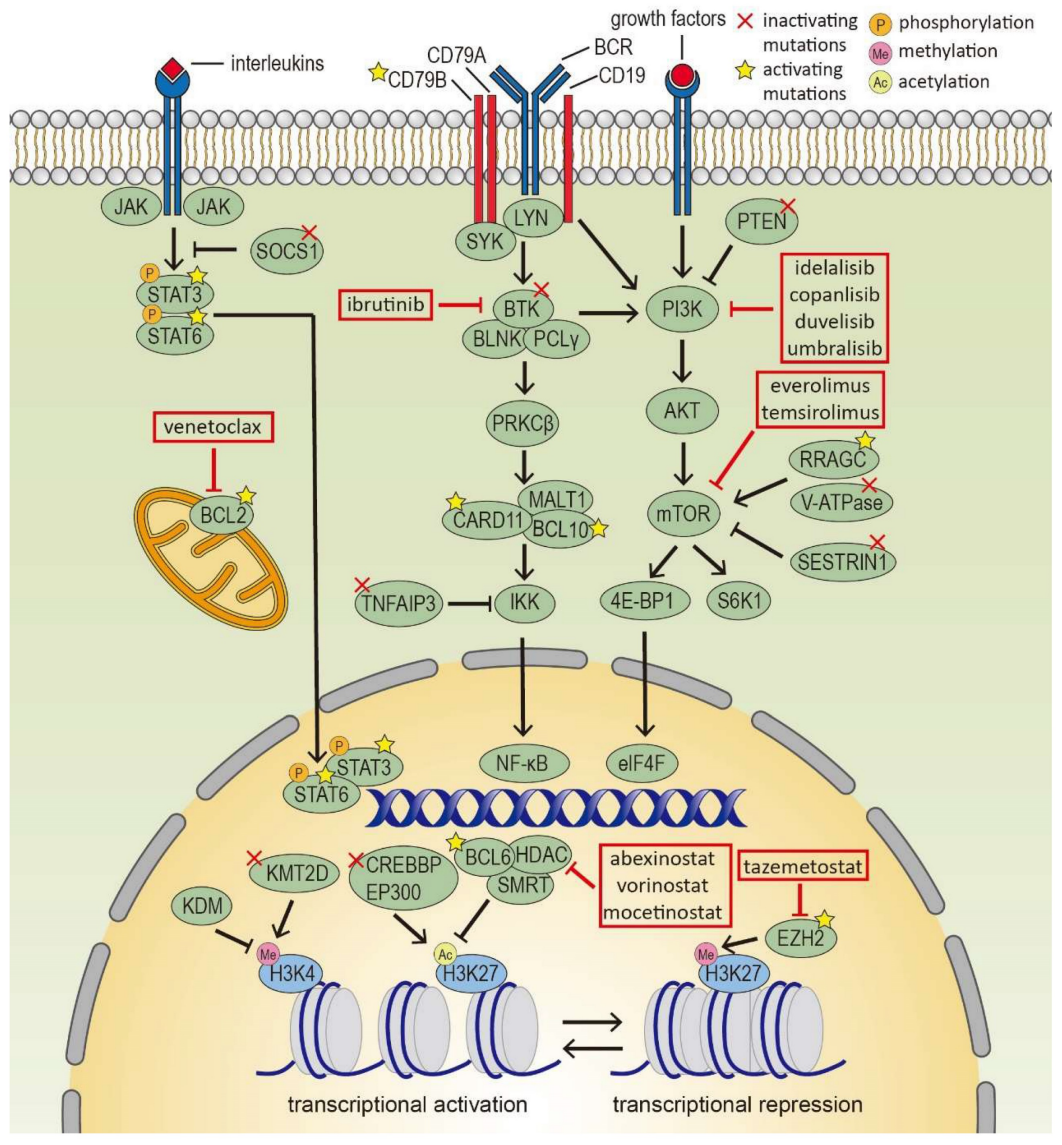
** Epigenetic modifiers and signaling pathways in follicular lymphoma cells.** Several epigenetic modifiers and signaling pathways are deregulated in FL cells, such as histone modifiers, BCR-NF-κB, JAK-STAT and PI3K/AKT/mTOR pathway, which serve as targets for novel precision therapies.

Aberrant activating or inhibitory mutations in oncogenic pathways in FL include BCR-NF-κB (BTK, CARD11, TNFAIP3, IGV region), JAK-STAT (SOCS1, STAT6), PI3K/AKT/mTOR (RRAGC, ATP6V1B2, VMA21, SESTRIN1) 42 (Figure 1). Besides, mutations in the NOTCH pathway, S1PR2-Gα13 axis, and cell cycle regulators are also detected. All of these contribute to the prolonged survival and sustained proliferation of FL cells.

About half of FL patients harbor mutations in genes associated with the BCR-NF-κB pathway. Most FL cells express IgM and mediate antigen-independent BCR signal that promotes the survival and proliferation of B cells. Activating mutations in CARD11 can initiate a spontaneous, receptor-independent activation of downstream NF-κB signaling 43. Inactivating mutations in BTK upregulate AKT phosphorylation 44. Loss-of-function mutations in TNFAIP3 activate NF-κB function 45. Besides, a BCR N-glycosylation sites asparagine-X-serine/threonine (N-gly sites) motif is present in over 80% of patients, which can interact with lectins in the TME and activate downstream BCR pathways 46. Interestingly, N-gly sites are early events in FL which are conserved during diagnosis, progression and transformation. N-gly sites provide a clear survival advantage for FL cells and are rarely found on normal B cells, suggesting therapeutic potential 46.

The JAK-STAT pathway genes are mutated in about 20% of patients. It can be activated by IL-4, IL-10 and IL-21 in the microenvironment, leading to downstream phosphorylation of STAT6 and STAT3 47. Activating mutations in STAT6 increase nuclear residency, and subsequently induce the expression of responsive genes CISH, FCER2, NFIL3 and CCL17 48. Inactivating mutations in SOCS1, an inhibitor of STAT, leads to the dysregulation of STAT activity 49.

The PI3K/AKT/mTOR pathway genes are mutated in 25% of patients, regulating cell growth and nutrient sensing. Mutated RRAGC proteins make mTORC1 insensitive to amino acid deprivation, enhance B-cell response and accelerate lymphomagenesis 50-52. ATP6V1B2 mutations activate mTOR, allowing cells to survive in low albumin concentrations 53. VMA21 mutations impair lysosomal function and lead to compensatory autophagy activation, a process that can be blocked by autophagy-inhibiting compounds 54. SESTRIN1 can be genetically inactivated or epigenetically silenced by EZH2, which affects p53-mediated regulation of mTORC1 and induces mRNA translation under genotoxic stress. Interestingly, mutations in EZH2 and RRAGC and loss of SESTRIN1 display mutual exclusivity, indicating that mTORC1 insufficiency can be generated by various mechanisms, and justifies the use of mTOR inhibitors 55.

Mutations in other oncogenic pathways are also observed in FL. NOTCH pathway is mutated in 18% of patients 56. Loss-of-function mutations in GNA13 of the S1PR2-Gα13 axis can be found in 10% of patients, promoting the dissemination, survival and SHM frequency of FL cells 57, 58. Nearly half of FLs have retinoblastoma (RB) pathway disruptions, including deletions of p16/CDKN2A/N and RB1, and gains of CDK4 19, 59, 60.

### Microenvironment modulators

A number of somatic mutations not only affect tumor cells, but also modulate the microenvironment development. These microenvironment modulators include TNFRSF14, CTSS, TNFAIP3, CREBBP, EZH2 and RRAGC. Depending on the effect on tumor-supporting Tfh cells, these genes exhibit two strategies of TME reprogramming. Mutations in TNFRSF14, CTSS, and TNFAIP3 recruit more Tfh cells to support FL proliferation. Loss-of-function mutations in TNFRSF14 (HVEM) disrupt normal interaction with inhibitory receptor BTLA expressed on Tfh cells, leading to higher Tfh infiltration and IL-4 activity 61. Activating mutations in CTSS increase MHC-II presentation and CD4+ T cell recruitment 62, 63. Inactivating mutations in TNFAIP3 exhibit a higher frequency in Th1 cells and CD8+ T cells 64. On the other hand, mutations in CREBBP, EZH2, and RRAGC reduce the frequency of infiltrating Tfh cells. Inactivation of CREBBP reduced MHC-II expression and antigen presentation, leading to decreased Tfh cells and cytotoxic T cell infiltration 65. Activating mutations in EZH2 downregulate MHC expression and limit the interaction of FL cells with Tfh cells 66, 67. Activating mutations in RRAGC are associated with decreased abundance of Tfh cells 68. Both RRAGC and CTSS mutations were mutually exclusive with TNFRSF14 mutations, suggesting the divergent evolution regarding dependence on TME 50, 63.

### Transcriptional heterogeneity

Single-cell RNA sequencing provided insights into the transcriptional heterogeneity both inter- and intra-patient. Genes differentially expressed among patient samples are mainly related to BCR and NF-κB signaling 69. In single patient lymph node biopsies of different locations, cluster analysis identifies marked site-to-site diversity in the expression profile of NF-κB signaling, antigen presentation, MAPK pathway, and cell adhesion, which indicate spatial heterogeneity within individual patients 46, 69-73. Therapeutic targets HDAC9 and CD79B are also differentially expressed, which could affect response to HDAC inhibitors or CD79B-directed therapies 69. BCR phylogenetic trees suggest that FL cells can migrate freely *in vivo*, undergo divergent evolution and independently develop between sites 46, 69. Overall, the degree of site-to-site variations in transcriptome profiles is consistent with BCR sequence variations.

Single-cell RNA sequencing also elucidates the diverse functional status of FL cells in the GC region within a single lymph node. FL cells present a continuous gene expression profile across the dark zone (DZ)/light zone (LZ) phenotype. In addition to conventional LZ and DZ phenotypes, approximately 30% of FL B cells exhibit a transitional state 74-76. Besides, FL cells exhibit GC desynchronization, highlighted by different co-expression patterns at the single-cell level 74. Furthermore, FL cells share a heterogeneous differentiation state across PC-, GC- and Mem-like B cells, with a small proportion exhibiting plasma cell-like features, while the majority span a continuous state between proliferating GC-like and quiescent Mem-like states 75. In single biopsies, transcriptional heterogeneity is independent of evolutionary subclones marked by BCR sequence 74.

Together, the simultaneous presence of multiple phenotypes suggests that FL cells may dynamically alternate between states. Epigenetics and TME can act as potential regulators in this process, paralleling normal GC reactions. Whether the functional status by transcriptome can reflect evolution pattern by the BCR sequence is still unclear. Further efforts are required to precisely define the high-risk populations in heterogeneous FL subclones for targeted therapies.

## Composition and function of TME in FL

The TME consists of T cells, macrophages, stromal cells, and a smaller proportion of neutrophils and natural killer (NK) cells 8, 77-79. Recently, single-cell RNA sequencing, mass cytometry by time of flight (CyTOF) and reverse phase protein array (RPPA) studies provide a comprehensive picture of microenvironmental cell components at the transcriptomics and proteomics levels 69, 71, 72.

### T cells

Tumor-infiltrating T cells (TILs) can be classified into three main populations, Tfh, Treg and CD8+ T cells. A prominent feature of FL TME is the higher concentration of CD4+ Tfh and Treg cells. TILs can also be divided into distinct subgroups based on functional distribution, including naive, effector, exhausted and memory cells 69, 71, 72, 80, 81. CYTOF studies revealed that TILs are skewed towards an exhausted phenotype in FL, marked by high expression of inhibitory checkpoints such as PD-1, LAG-3 and TIGIT, as well as low expression of co-stimulatory receptors CD27 and CD28, leading to reduced interaction with antigen-presenting cells (APCs) and impaired TCR signaling responses 73, 80, 82-87. However, the expression of immune checkpoints does not fully represent a defective state, since PD-1 is also highly expressed in active Tfh cells 47, 84. CYTOF studies also identified that specific subpopulations of PD-1(+) T cells and deficient CD4(+) memory T cells are related to poor prognosis 80. Besides, a recent study has defined four FL TME subtypes based on T-cell subsets population, in which a T cell-depleted subset exhibited an inferior outcome 88.

Tfh cells are necessary for the selection and differentiation of B cells in normal GC, and are marked by a CD4+CXCR5+ICOS+PD1+BCL6+ immunophenotype. FL Tfh maintain a high production of cytokines and chemokines such as IL-4, IL21, TNFα, IFNγ, and CXCL13. IL4 and IL21 activate STAT6 in FL B cells, supporting FL growth and survival. IL-4 also drives macrophages toward the M2 phenotype and triggers upregulation of CXCL12 in lymphoid stromal cells (LSCs) 89, 90. RPPA studies verified high concentrations of IL-4 and the elevated downstream phosphorylation of STAT6 and ERK in FL at the protein level 91. Besides, Tfh highly expresses CD40L, IL6R and CTLA. CD40L binds to CD40 on FL B cells, activates the NF-κB signaling pathway, and induces FL B cells to respond to macrophage-derived IL-15 92-94. Interestingly, single-cell analysis revealed that Tfh abundance is the only microenvironmental cell subpopulation that correlated with FL spatial heterogeneity, and is associated with CD40 expression, reinforcing the role of Tfh in FL development 69.

Treg cells are characterized by a CD4+CD25+FOXP3+ immunophenotype with a frequent expression of CD25, GITR, CTLA-4 and CD45RO 85, 95. FL B cells convert conventional T cells into Treg, recruit and promote the proliferation of Treg through a variety of chemokines (CXCL13, CCL17, CCL19 and CCL22) and membrane molecules (CD70, CD80, CD86 and ICOSL) 85. Treg cells inhibit CD4+ T cell proliferation and CD8+ T cell cytotoxic function 82, 85. Notably, in addition to classical Treg, a population of CXCR5+PD-1+ICOS+ T follicular regulatory (Tfr) cells was found. Tfr cells inhibit Tfh and B cell activation, suggesting their potential to exert antitumor effects 96-98.

CD8+ T cells are the major population that exerts specific cytotoxic antitumor immunity 99 CYTOF and CITE-seq analysis demonstrated that CD8+ T cells in FL are skewed towards effector cells which exhibit an enhanced capacity to produce granules (granzyme B and perforin) and cytokines (IFNγ and TNFα). However, these cells are more immunologically depleted due to sustained antigenic stimulation, marked by high expression of PD-1, TIGIT, TIM-3, ICOS, BTLA, and LAG3 82, 100, 101. Besides, FL CD8+ T cells show downregulated TCR-induced ERK phosphorylation and IFNγ production, while TCR proximal signaling is not affected 86. Furthermore, PI3K inhibitors downregulate transcription factors responsible for effector cell differentiation, indicating a therapeutic mechanism of PI3K inhibitors on the TME 100.

### Macrophages

Depending on the stimulatory signals received, macrophages can be divided into M1 and M2 subtypes, and are generally polarized to M2 subtypes in tumors 102. M2 macrophages express DC-SIGN, which binds to the N-glycosylation site on the BCR of malignant B cells to promote cell survival 103. M2 macrophages also produce various cytokines to enhance angiogenesis (VEGF and angiopoietin), recruit monocytes and neutrophils (CCL2, CLL3 and IL-8), and promote dissemination (CXCL12, CCL18 and MMP9) 104. Moreover, phagocytosis by macrophages is hampered by the interaction of SIRPα with the "do not eat me" receptor CD47, which is abundantly expressed on FL B cells. Using CYTOF, FL macrophages can be further divided into three subpopulations based on the expression levels of SIRPα and CD14 ((CD14(+)SIRPα(hi), CD14(-)SIRPα(low), and CD14(-)SIRPα(neg)), which exhibit distinctive differentiation, migration, immune function and prognostic values 105.

### Stromal cells

Stromal cells support the survival and dissemination of tumor cells, and are involved in the reprogramming of the immune compartment. In FL, malignant B cells reprogram LSC precursors into cancer-associated fibroblasts (CAFs) through expressing CCR7, CXCR4, TNF, LT and TGF-β. FL stromal cells overexpress a variety of chemokines such as CXCL12, CCL19 and CCL2 79. The two most prominent stromal cell subpopulations are FRCs and FDCs. FRCs are present in the T-cell region around the follicle and secret extracellular matrix 106. FRCs contribute to the polarization of FL-Tfh by enhancing IL-4 secretion in a Notch- and ICAM1/LFA1-dependent manner 106. FRCs also promote B cell activation and adhesion through the expression of CXCL12, CXCL13, IL-7 and BAFF, which can be antagonized by BTK and PI3K inhibitors 79, 90. FDCs are specialized APCs enriched in the B-cell zone. The FDC network is often disrupted compared to the FRC network in FL, and malignant B cells reduced expression of the GC-confinement receptors S1PR2 and P2RY8, suggesting a less role for FDCs to support FL B cells 79, 107. FDCs activate B cells through presenting lectins or antigens to BCR. Besides, FDCs express TIGIT ligand, reinforcing their immunosuppressive function 82, 86.

A recent study classified LSCs into four major clusters by expression of CD49a (ITGA1), podoplanin (PDPN) and CD21, including cell populations of FRCs (PDPN+CD21-CD49a+), FDCs (PDPN+CD21+CD49a-), double-negative (DN) perivascular cells (PDPN-CD21-CD49a+), and a less defined fourth subset (PDPN+CD49a-). DNs are similar to cells with precursor capabilities, expressing nestin and markers of adipocytes and osteoblasts, and can differentiate toward FRC-like cells in response to TNF and LT 79. By single-cell clustering analysis, lymph node stromal cells can also be divided into blood endothelial cells (BECs), lymphatic endothelial cells (LECs), and non-endothelial SCs (NESCs). Upregulation of a number of previously unidentified genes and interaction pairs such as CD70-CD27 was observed 78. Using histology examination, the location and quantification of stromal cells was assessed *in situ* and showed higher abundance than common sequencing methods 12.

Together, these results indicate that the heterogeneity of microenvironment cells lies far beyond our understanding and remains to be further functionally explored. The cellular components of TME in the bone marrow are very different from those in lymph nodes (reviewed in separate articles 108, 109). FL subclones with different gene expression profiles are detected between bone marrow and lymph nodes 109, 110. Currently no systematic study has combined the relationship between TME components and genomic and transcriptomic abnormalities of FL B cells to study how TME contributes to the spatial heterogeneity between lymph node sites and extranodal involvement. In addition, it remains to be elucidated how FL-TME interactions alter throughout the course of the disease. A better understanding of how specific TME components regulate the onset, progression, therapeutic response and relapse of FL would provide novel opportunities for harnessing TME as treatment options and potential biomarkers.

## POD24 and transformed FL: underlying mechanism and biomarkers

The clinical course and outcomes of FL are heterogeneous. A subset of patients who develop early disease progression within 24 months of diagnosis (POD24) or histological transformation has a poor prognosis. Approximately one-third of POD24 patients also undergo transformation 111, 112. The mechanisms underlying these high-risk FL populations are not yet fully understood. Prognostic models remain to be explored to identify these patients in order to provide personalized therapeutic interventions.

As to genomic aberrations, comparative analysis of paired samples revealed an increase in genomic complexity in patients with transformed FL (t-FL) and POD24, including mutations and copy number variations (CNVs). t-FL is especially enriched in genetic aberrations involving the NF-κB pathway (MYD88, TNFAIP3), DNA damage responses (TP53), transcription factors (EBF1, MYC, IRF4), immune surveillance (CD58, B2M), cell-cycle (CDKN2A/B, CCND3) and dissemination (S1PR2, GNA13). t-FL also shows a dysregulation of miRNA (miR-142, miR-150, miR-7e-5p) and a higher level of SHM 14, 16-20, 113-117. About 80% of t-FL is of the GC B-cell-like (GCB) subtype and about 16% is of activated B-cell-like (ABC) subtype. ABC subtype has a lower incidence of t(14;18) and increased mutations in BCR-NF-κB signaling 18, 19. For POD24 patients, single-cell RNA sequencing identified the upregulation of BCR signaling pathway 12. The overall SHM burden in samples from early versus late/never progressors is not significantly different. A ten-gene list was found to be mutated more commonly in POD24 patients, including KMT2C, TP53, BTG1, MKI67, XBP1, SOCS1, B2M, FAS, IKZF3 and MYD88. Gains in VRK2 and FANCL by small insertions were also observed. About 80% of POD24 patients had mutations in any of the ten genes despite low mutation frequencies, suggesting a role of rare genetic events in FL progression 19, 115. Notably, a clonal evolution study shows distinct patterns underlying transformation and early progression, in which t-FL is generated from clones that are rare or absent in diagnostic samples, while POD24 arisen from detective treatment-resistant clones that already exist at diagnosis 19.

The prognostic value of FL TME is also identified by immunohistochemistry, flow cytometry and RNA sequencing. TME factors associated with t-FL include a low lymphocyte-to-monocyte ratio (LMR), diffuse pattern of PD1+ T cells and CD14+ FDCs, follicular distribution of FOXP3+ cells, and increased vessel density 118-122. POD24 events are significantly enriched in patients with low immune infiltration level marked by low expression of the immune checkpoint PD-L2 123. Fibroblasts, DC-SIGN+ cells and extracellular matrix (ECM) were found enriched in early progression FL patients, as verified by quantitative imaging.^12^ Other TME predictors for POD24 include lack of intrafollicular CD4 expression as well as low expression of CD8+ T-cell markers and genes regulating Th1 and Th2 responses 81, 124.

Prognostic models of FL regarding clinical factors include FLIPI1, FLIPI2, PRIMA-PI and FLEX, which are designated based on different indexes and treatment regimens 125-128. Integrated models that combine clinical and molecular factors have also been proposed, including m7-FLIPI, POD24-PI and a 23-gene model 34, 129, 130. The sensitivity and specificity of these models in predicting POD24 range from 43% to 86%, with the highest sensitivity for POD24-PI (78%) and specificity for m7-FLIPI (86%) 129, 131. t-FL is also found relative to a range of clinical factors, including high histological grade and high FLIPI score, while the response to therapy had no impact on the risk of transformation 112.

To date, no genetic, microenvironmental, clinical or combined models have shown adequate efficiency as biomarkers for t-FL or POD24 to direct clinical practice. The integration of multi-omics data is hopeful to incorporate multiple levels of information for detailed patient stratification and accurate prediction.

## Novel approaches targeting multi-omics alterations

With a better understanding of FL and its microenvironmental, significant progress has been made in FL treatment approaches. New targeted agents and immunotherapies offer alternatives for relapsed/refractory (R/R) patients following traditional rituximab-based chemoimmunotherapy (Figure 1).

Since epigenetic modifiers deregulations are key events in FL, epigenetic therapies play an important role in FL treatment. EZH2 inhibitor tazemetostat demonstrated favorable clinical response with an ORR of 69% in EZH2 mutant patients and 35% in EZH2 wild-type patients, with a median PFS of 13.8 and 11.1 months. EZH2 inhibitor is well tolerated, with only 4% of patients showed serious adverse effects 132. Tazemetostat has received accelerated approval from the FDA, and its combination therapies with CD20 monoclonal antibodies or immunomodulatory agents are also in clinical trials 133. Inactivation of CREBBP and KMT2D are difficult to target directly, but can be rescued by inhibiting their antagonists. Histone deacetylase 3 (HDAC3) inhibitors (abexinostat, vorinostat, mocetinostat) compensate for CREBBP/EP300 deficiency by targeting the BCL6 /SMRT/HDAC3 complex 25, 134-137. KDM5 inhibition can restore histone methylation and the expression of genes repressed by KMT2D loss 138. Other epigenetic agents include DNMT inhibitors, PRMT inhibitors and BET inhibitors, which have shown efficacy in other hematological malignancies such as acute myeloid leukemia and diffuse large B-cell lymphoma, but have not yet been implemented in FL 133, 139.

BCL2 overexpression is considered a major driver of FL, but the BCL2 inhibitor venetoclax failed to show a significant benefit in monotherapy or combination with rituximab/bendamustine-rituximab 140. The reasons could be BCL2 mutations, heterogeneity of BCL2 expression among subclones, expression of other anti-apoptotic genes, and a decrease in BCL2 dependence at advanced stages of the disease 141. Similarly, although BCR signaling-related genes are highly activated in FL, the effect of the BTK inhibitor ibrutinib was unsatisfactory with ibrutinib monotherapy showing ORR and CR rates of 21-38% and 11-13% 142, 143. Possible explanation is an alternative BCR pathway independent of BTK 144. Other BTK inhibitors (acalabrutinib and zanubrutinib) and SYK/JAK inhibitor cerdulatinib are under testing in single agent or combination trials 145-147.

The PI3K-AKT-mTOR pathway is highly activated in FL. Four PI3K inhibitors (Idelalisib, Copanlisib, Duvelisib, Umbralisib) have been approved with varying efficacy and toxicity profiles, depending on the specific subunit targeted 148-152. The four agents showed moderate activity with ORR ranging from 42% to 59% and the median PFS about 10 months. Clinical trials for other PI3K inhibitors and combination regimens are ongoing 133, 153, 154. Besides, mTORC1 inhibitors everolimus and temsirolimus exhibited an ORR of 61% and 54% in R/R indolent lymphoma 155, 156.

New immunotherapy approaches that harness TME to activate anti-tumor immunity are being explored and have shown promising potential, including immune modulators, immune-checkpoint inhibitors, bispecific antibodies (BSAbs) and chimeric antigen receptor (CAR) T cell therapy.

Lenalidomide is a non-specific immunomodulatory agent that binds cereblon, induces degradation of transcriptional factors, and increases the cytotoxicity effect of CTLs, NKs and antibodies 157. The combination of lenalidomide plus rituximab (R2) significantly improved PFS for R/R FL than rituximab alone, and R2 has been approved as the standard of care for the relapsed setting 158-160. In untreated FL patients, R2 shows comparable efficacy to conventional immunochemotherapy as the first-line treatment of FL 159. A similar high response rate can also be observed in lenalidomide and obinutuzumab 161, suggesting the chemo-free regimen can be a less-toxic first-line option for FL patients to avoid the side effects of chemotherapy.

Despite the enrichment of PD-1+ TILs in FL TME, the effect of PD-1 inhibitors (nivolumab and pembrolizumab) in FL has been disappointing. In a phase II study of R/R FL, nivolumab only had an ORR of 4% and median PFS of 2.2 months, and the response had no correlation with PD-1 or PD-L1 expression 162, 163. The efficacy of nivolumab was improved when combined with ibrutinib, with an ORR of 33% 162. Combining Urelumab, an agonist of co-stimulatory receptor 4-1BB (CD137), fails to enhance clinical activity compared to rituximab alone 164. Other immune checkpoints such as LAG-3, TIM-3, and TIGIT are also highly expressed in FL TILs, and relative combination therapies are under investigation 165-167.

The phagocytosis of macrophages can be suppressed by CD47 on tumor cells that bind with SIRPα on macrophages 168. In a phase Ib/II study, CD47 monoclonal antibody Magrolimab (Hu5F9-G4) in combination with rituximab induced an ORR and CR rate of 71% and 43% in 7 patients with R/R FL 169. Other agents of interest to target macrophages include ALX148, TTI-622, and CD19-CD47 bispecific antibodies 154.

Bispecific antibodies (BSAbs) and autologous CAR-T cells generate immune responses by targeting B cell markers on the surface of lymphoma cells. BSAbs consists of two binding domains, usually CD3 for T cells and CD20 or CD19 for lymphoma cells. A variety of BSAbs has shown remarkable efficacy in clinical trials, including mosunetuzumab, odronextamab, epcoritamab, and glofitamab, with ORR of 64%-93% 170-175. CAR-T cells are engineered T cells that can specifically recognize and eradicate cells expressing particular antigens. Based on the ZUMA-5 trial, axicabtagene ciloleucel, a CD19 CAR-T therapy, has been approved for R/R FL with ORR and CR rates of 94% and 60% 176, 177. The ELARA study of tisagenlecleuce has a similar high ORR of 86% and a CRR of 66% 178, 179. In addition, a series of clinical trials combining CAR-T with other targeted agents and BSAbs are under evaluation. Serious side effects for CAR-T and BSAbs therapies are cytokine release syndrome and neurological toxicity, which will require further product optimization and adjuvant drug application. Long-term data are needed to support the durability of these therapies. Due to the impressive efficacy demonstrated in clinical trials, the future of immunotherapies is highly promising.

Despite the wide range of available treatments for FL patients, from first-line rituximab-based regimens (including R-CVP, R-CHOP, RB and R2) to a multitude of targeted agents and immunotherapies, there is currently no uniform standard for optimal sequencing and combination of therapies for the individual. There are very limited biomarkers to predict patient response except for EZH2 inhibitors. In addition, the mechanism of action of many agents is not limited to their known targets, particularly with effects on TME. Tazemetostat can enhance T-cell recruitment through modulating MHC II and chemokine expression 180. HDAC inhibitors induce expression of IFN pathway and antigen-presenting genes, and work synergistically with checkpoint inhibitors to restore the cytotoxic ability of T cells 181. Idelalisib can reduce the recruitment of Tfh and Treg cells, inactivate the suppressive activity of Treg cells, impair FDCs proliferation, and restore FL response to venetoclax 182, 183. Activity of CD47 inhibitors also involves DCs and CTLs 184. Response to PD-1 blockade is associated with NK cells and CD4+ T cells 185. Therefore, further studies on the mechanisms of action can help to better understand the drivers of response and resistance for patients. In addition, the potential alternative mechanism is thought to possibly interfere with the efficacy of other therapies, and further clinical trials are needed to confirm the efficacy of combination and sequential therapy in practice.

## Prospects

Multiple approaches can be applied to unravel key molecular mechanisms that contribute to FL. Correlation analysis between transcriptome and genome or epigenome can help identify differentially regulated pathways that are subject to gene mutations or epigenetic modifications. Integrating proteomic data with other omics can reveal functional consequences of upstream alterations, as well as the relationship between intracellular abnormalities and FL-TME crosstalk. In addition, network analysis forms a web of interactions between genes, proteins and other molecules. Machine learning can analyze large-scale datasets to generate potential biomarkers and personalized therapy through new algorithms. With these knowledge, subsequent experimental validation can be performed to elucidate primary alterations and secondary functional effects. Therefore, the integration of multiple omics provides a more comprehensive view of FL malignant cells and the interaction with TME, leading to the development of novel diagnostic and therapeutic strategies.

## Conclusion

FL is an indolent but still incurable disease. Over the last decade, the understanding of the abnormalities in FL and its microenvironment has been dramatically improved. The multi-omics analysis provides a panorama view of the genome, epigenome, transcriptome, and microenvironment abnormalities of FL. Future research is required to elucidate how these complex factors work together to promote disease development and predict therapeutic responses. The combination of molecular subtype with clinical heterogeneity will lead to precise risk stratification and personalized therapy in FL.

## Figures and Tables

**Table 1:** Representative genetic aberrations in FL.

Gene	Frequency (%)	Alterations	Function	Refs
***Epigenetic modifiers***				
KMT2D	70-90	LOF	Histone methyltransferase	15,16,20,21,34,66
CREBBP	50-70	LOF	Histone acetyltransferase	
EP300	20-30	LOF	Histone acetyltransferase	
EZH2	20-30	GOF	Histone methyltransferase	
Linker histones	27-44	LOF	linker histones; chromatin remodelling	
ARID1A	11-15	LOF	SWI/SNF complex; chromatin remodelling	
BCL7A	10	LOF	SWI/SNF complex; chromatin remodelling	
Transcription factors				
BCL6	Mutations, 5 Translocations, 20	GOF	Transcription factors	15,16,17,18,20,21,34
MEF2B	7-15	GOF		
FOXO1	12	GOF		
EBF1	17	unknown		
IRF8	6-13	unknown		
IRF4	11	unknown		
***BCR-NF-κB pathway***				
CARD11	11	GOF	Signals transducer downstream BCR activation	16,20,21,44,45,48
BTK	7-10	LOF	Tyrosine kinase	
TNFAIP3	10	LOF	Inhibitor of NF-κB, immune response modulator	
CD79B	10	GOF	BCR complex	
IGV	80	GOF	N-glycosylation of IGV region of BCR	
***JAK-STAT pathway***				
STAT6	12	GOF	Signal transduction transcription activation	16,20,48,49
SOCS1	8-10	LOF	JAK-binding protein	
***PI3K/AKT/mTOR pathway***				
RRAGC	10-20	GOF	Guanine nucleotide-binding protein	20,52,53,54,55
ATP6V1B2	10-22	LOF	V-ATPase complex	
ATP6AP1	10	LOF	V-ATPase complex	
VMA21	12	LOF	V-ATPase complex	
SESTRIN1	Deletion, 20	LOF	Negative regulator of mTOR activity	
***NOTCH pathway***				
NOTCH1, NOTCH2, NOTCH3, NOTCH4, DTX1, SPEN	18	LOF	NOTCH pathway	21
***Migration***				
GNA13	10	LOF	Guanine nucleotide-binding proteins	18,19,20,52
***Cell cycle***				
RB1	Deletion, 12	LOF	Cell cycle regulator	20,59
CDK4	Copy number gain, 29	GOF		
CCND3	15	GOF		
***Survival***				
BCL2	Mutations, 50Translocations, 85	GOF	anti-apoptosis	25,26
***Immune evasion***				
TNFRSF14	20-50	LOF	Receptor	16,20
CTSS	4-22	GOF	Protease	62,63
EPHA7	70	LOF	Receptor	22

These are mutations unless specifically mentioned as translocations or copy number variations. GOF: gain-of-function LOF: loss-of function
